# Acute respiratory morbidity in late preterm infants

**DOI:** 10.1186/1824-7288-40-S2-A34

**Published:** 2014-10-09

**Authors:** Simonetta Picone, Roberto Aufieri, Piermichele Paolillo

**Affiliations:** 1 Division of Neonatology and Neonatal Intensive Care, Department of Maternal and Child Health, Casilino General Hospital, Roma, Italy

## Background

Late preterm (LP) infants [gestational age (GA): 34-36 weeks] are at increased risk of neonatal acute respiratory morbidity compared with term infants (GA: 37- 41)[1,2]. The observed rate of acute respiratory morbidity, in a population of about 20,000 LP infants, was 10-12% vs 1.4% of term infants [1]. Transient tachypnea of the newborn (TTN) and respiratory distress syndrome (RDS) are the most common diagnosis, with RDS rate reaching 10.5% in infants born at 34 weeks of GA [1,2]. Major causes of respiratory morbidity in LP are: prematurity and birth by Cesarean Section (CS) [1,3].

## Material and methods

We retrospectively studied 830 LP and moderate preterm (MP) infants (GA: 33-36 weeks) admitted to our unit from June 2009 to December 2013. Infants were classified according to GA: 33 weeks (n=129), 34 weeks (n=176), 35 weeks (n=225), 36 weeks (n=300). Clinical charts for each patient were reviewed and main diagnosis recorded.

## Results

Twenty-six percent of LP/MP infants (214/830) had an acute respiratory disorder. The most frequent diagnosis were: TTN (n=75; 9.0%); respiratory failure (RF) (n=65; 7.8%) and RDS (n=62; 7.5%); pneumothorax / pneumomediastinum (n=16; 1.9%); pneumonia (n=13; 1.6%); apnea of prematurity (n=5; 0.6%); persistent pulmonary hypertension (n=2; 0.2%). All the 62 infants with RDS were intubated, required mechanical ventilation (1-4 days) and surfactant administration (1-4 doses). The forty-three percent of infants with RDS also had a concomitant diagnosis of infections. The infection rate in infants with RDS was significantly higher than that in other respiratory morbidities (p<0.05). Complete results are reported in Table 1. Of the 62 cases of RDS reported: 60 resolved and 2 deceased (one patient with necrotizing enterocolitis, one patient with disseminated intravascular coagulation).

**Table 1:** Acute respiratory morbidity in LP/MP infants (n= 830)

	GA^a^ (weeks)				
	
	33	34	35	36	TOT
**n**	129	176	225	300	830

**Respiratory morbidity**	60 (46.5)	53 (30.1)	42 (18.7)	59 (19.7)	214 (25.8)

**TTN^b^** n (%)	14 (10.9)	15 (8.5)	16 (7.1)	30 (10.0)	75 (9.0)
**RF^c^** n (%)	31(24.0)	14 (8.0)	12 (5.3)	8 (2.7)	65 (7.8)
**RDS^d^** n (%)	12 (9.3)	24 (13.6)	12 (5.3)	14 (4.7)	62 (7.5)
**PNX^e^** n (%)	1 (0.8)	-	8 (3.5)	7 (2.3)	16 (1.9)
**Pneumonia** n (%)	1 (0.8)	3 (1.7)	1 (0.4)	8 (2.7)	13 (1.6)
**AOP^f^** n (%)	3 (2.3)	-	-	2 (0.7)	5 (0.6)
**PPH^g^** n (%)	-	1 (0.6)	1 (0.4)	-	2 (0.2)
**MAS^h^** n (%)	-	-	-	-	-

					

**Infection in infants with RDS** n (%)	3 (25.0)	9 (37.5)	6 (50.0)	9* (64.3)	27* (43.5)

**Infection in infants with other respiratory morbidity** n (%)	8 (16.7)	10 (34.5)	11 (39.7)	14* (31.1)	43* (28.3)

* p<0.05

a gestational age. b transient tachypnea of newborn. c respiratory failure. d respiratory distress syndrome. e pneumothorax / pneumomediastinum. f apnea of prematurity. g persistent pulmonary hypertension. h meconium aspiration syndrome.

## Conclusions

Acute respiratory morbidity in our unit affects a quarter of LP/MP infants. An important percentage (7.5%) is represented by RDS, that is often associated with infection. Infants born at 34 weeks of GA are the population at higher risk of RDS. Even if rate and severity of acute respiratory morbidity in LP are already described by a number of epidemiological studies, further investigation is needed to better clarify the optimal timing and dose of surfactant administration and to correlate different strategies of respiratory management with long-term respiratory and neurological outcomes. The high infection rate found among infants with RDS and acute respiratory morbidity, emphasizes the importance of a prompt diagnosis and treatment of chorioamnionitis and perinatal infections.
